# Viral Mimicry of Cdc2/Cyclin-Dependent Kinase 1 Mediates Disruption of Nuclear Lamina during Human Cytomegalovirus Nuclear Egress

**DOI:** 10.1371/journal.ppat.1000275

**Published:** 2009-01-23

**Authors:** Sofia Hamirally, Jeremy P. Kamil, Yasmine M. Ndassa-Colday, Alison J. Lin, Wan Jin Jahng, Moon-Chang Baek, Sarah Noton, Laurie A. Silva, Martha Simpson-Holley, David M. Knipe, David E. Golan, Jarrod A. Marto, Donald M. Coen

**Affiliations:** 1 Department of Biological Chemistry and Molecular Pharmacology, Harvard Medical School, Boston, Massachusetts, United States of America; 2 Dana-Farber Cancer Institute, Harvard Medical School, Boston, Massachusetts, United States of America; 3 Cardiovascular Division, Brigham and Women's Hospital, Harvard Medical School, Boston, Massachusetts, United States of America; 4 Department of Microbiology and Molecular Genetics, Harvard Medical School, Boston, Massachusetts, United States of America; 5 Hematology Division, Brigham and Women's Hospital, Harvard Medical School, Boston, Massachusetts, United States of America; Oregon Health and Science University, United States of America

## Abstract

The nuclear lamina is a major obstacle encountered by herpesvirus nucleocapsids in their passage from the nucleus to the cytoplasm (nuclear egress). We found that the human cytomegalovirus (HCMV)-encoded protein kinase UL97, which is required for efficient nuclear egress, phosphorylates the nuclear lamina component lamin A/C *in vitro* on sites targeted by Cdc2/cyclin-dependent kinase 1, the enzyme that is responsible for breaking down the nuclear lamina during mitosis. Quantitative mass spectrometry analyses, comparing lamin A/C isolated from cells infected with viruses either expressing or lacking UL97 activity, revealed UL97-dependent phosphorylation of lamin A/C on the serine at residue 22 (Ser^22^). Transient treatment of HCMV-infected cells with maribavir, an inhibitor of UL97 kinase activity, reduced lamin A/C phosphorylation by approximately 50%, consistent with UL97 directly phosphorylating lamin A/C during HCMV replication. Phosphorylation of lamin A/C during viral replication was accompanied by changes in the shape of the nucleus, as well as thinning, invaginations, and discrete breaks in the nuclear lamina, all of which required UL97 activity. As Ser^22^ is a phosphorylation site of particularly strong relevance for lamin A/C disassembly, our data support a model wherein viral mimicry of a mitotic host cell kinase activity promotes nuclear egress while accommodating viral arrest of the cell cycle.

## Author Summary

Human cytomegalovirus (HCMV) causes life-threatening disease in transplant patients and people with AIDS, and is also an important cause of birth defects. Like all viruses, HCMV must have a way to leave the host cell, so that it can infect new cells. Moreover, as a member of the herpesvirus family, HCMV replicates its DNA in the nucleus, so it must have mechanisms to ensure that its genetic material can exit from the nucleus (nuclear egress). HCMV encodes a protein kinase, UL97, which is required for efficient nuclear egress. We found that UL97 aids nuclear egress by mimicking a host cell enzyme that normally helps break down a protein meshwork in the nucleus during cell division. The enzyme activity of UL97 pokes holes in the meshwork that allow nascent HCMV virions to gain access to the nuclear membrane. UL97 is also an important target for drugs for treating HCMV disease. This work not only helps explain how these drugs act, but also highlights the potential of targeting nuclear egress for the discovery of new drugs.

## Introduction

Human cytomegalovirus (HCMV) is a pathogen that is especially dangerous in immunocompromised individuals [1]. As is true for all viruses, HCMV replication depends on the interplay between viral and host cell functions. An important example of this interplay is nuclear egress, a stage during which herpesviral DNA-containing capsids (nucleocapsids) exit the nucleus [2]. An important obstacle for the exiting nucleocapsids is a meshwork underlying the inner nuclear membrane known as the nuclear lamina, whose principal components are intermediate-filament proteins known as lamins [3],[4]. There are two major classes of lamins in mammalian cells: A-type lamins, which comprise the four lamins encoded by alternative splicing from the *LMNA* gene, lamin A, AΔ10, C, and C2 (collectively lamin A/C), and B-type lamins (lamin B), which are encoded by the *LMNB1* and *LMNB2* genes. A major function of lamins is to help maintain the structure of the nuclear envelope. Accordingly, along with the nuclear envelope, the nuclear lamina must be disassembled during mitosis and then reassembled after mitosis. These dynamic processes are regulated by phosphorylation of lamins. In particular, it is well established that Cdc2/cyclin-dependent kinase (CDK) 1 disassembles nuclear lamina by phosphorylation of specific sites on lamins during mitosis [5],[6],[7]. CDK1 phosphorylation of lamin A/C at Ser^22^, and of lamin B at the equivalent position, have been shown to be especially crucial for lamina disassembly [5],[8]. It is thought that phosphorylation at this site interferes with head-to-tail interactions between lamins (reviewed in [3],[4]). HCMV arrests cells at the G1/S boundary during the cell cycle [9],[10],[11], and therefore is unable to utilize this normal pathway for dissolution of the nuclear lamina for nuclear egress. Interestingly, despite the G1/S arrest, CDK1 and cyclin B are upregulated in HCMV-infected cells [12],[13],[14]. However, these proteins do not appear to accumulate in the nuclei of infected cells to the extent seen in mitotic cells [14].

It has been proposed, initially from work on murine cytomegalovirus (MCMV), that a complex of two viral polypeptides (UL50 and UL53 for HCMV) recruits calcium-dependent protein kinases C (PKCs), to the nuclear envelope to phosphorylate lamins, disrupt the nuclear lamina, and permit nuclear egress [15],[16],[17]. There is evidence that PKC phosphorylation of lamins is important for dissolution of nuclear lamina (e.g. [18]). However, it has not been demonstrated that recruitment of PKC is sufficient or necessary to cause lamin disruptions during HCMV infection or to permit nuclear egress of HCMV. On the other hand, an unusual protein kinase, UL97, which is encoded by HCMV, has been shown to be required at the stage of nuclear egress for efficient replication of HCMV [19]. Some evidence for UL97 effects on nuclear lamina components has been presented [20]. Interestingly, it has recently been shown that UL97 has activities similar to those of CDKs (as hypothesized [21]), but is not subject to cellular inhibitors of CDK function [22]. UL97 is also a target for specific inhibition by a new antiviral drug, maribavir [23], which is currently in phase III clinical trials.

In this study, using purified UL97, we show that UL97 phosphorylates lamin A in vitro on Ser^22^, a site utilized by CDK1 to mediate lamin disassembly. In HCMV-infected cells, we detected UL97-dependent phosphorylation of lamin A/C at Ser^22^, and inhibition of phosphate incorporation into lamin A/C by maribavir. Finally, morphological alterations in the host cell nucleus and nuclear lamina during HCMV infection were observed to depend on UL97. Taken together, our data argue that UL97 directly phosphorylates lamin A/C during HCMV replication to promote lamin disassembly during nuclear egress.

## Results

### Identification of lamin A/C as a candidate substrate of UL97

To investigate the role(s) of UL97 during HCMV infection, we searched for candidate substrates of UL97 by implementing a proteomics strategy. Human foreskin fibroblast (HFF) cells infected with wild-type (wt) HCMV strain AD169 (multiplicity of infection (MOI) = 1), with a *UL97* deletion mutant (RCΔ97) [24], or with wt virus under conditions where UL97 was pharmacologically inhibited using maribavir [23] were radiolabeled with ^32^P orthophosphate, and the proteins were separated on 2-dimensional gels (Figure S1). Spots containing labeled phosphoproteins from wt-infected cells that differed from those from the other conditions were excised from the gels, digested with trypsin, and analyzed by liquid chromatography-tandem mass spectrometry (LC-MS/MS). A number of polypeptides were identified; these are tabulated in Table S1. The protein for which the most peptides were identified was lamin A/C, which is a major component of the nuclear lamina [3],[4]. Given that HCMV UL97 is important at the stage of nuclear egress [19] and that the nuclear lamina forms a barrier to nuclear egress, we hypothesized that UL97 phosphorylates lamin A/C to mediate nuclear egress.

### Purified UL97 phosphorylates lamin A *in vitro* on sites phosphorylated by CDK1

A previous report [20] described the phosphorylation of lamins in anti-FLAG immunoprecipitates from lysates of cells in which FLAG-UL97 was expressed by transfection, but only when lamins were simultaneously immunoprecipitated by anti-lamin antibodies. No controls in which lamins were immunoprecipitated, but UL97 was absent or inactive were shown. Thus, it was not demonstrated that UL97 directly phosphorylates lamins. Additionally, sites of phosphorylation were not reported.

**Figure 1:**
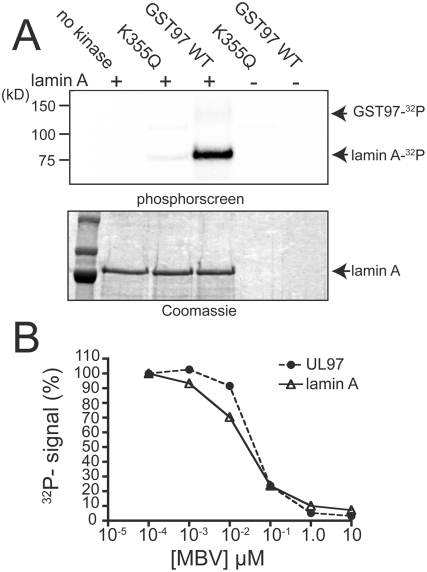
In vitro phosphorylation of lamin A by GST-UL97. (A) Recombinant His-tagged lamin A was incubated in kinase reaction buffer in the presence of γ-^32^P-ATP either alone (no kinase), with catalytically deficient GST-UL97 K355Q (K355Q), or with wild-type GST-UL97 (GST97 WT). GST-UL97 K335Q or wild-type GST-UL97 were also incubated in kinase buffer without lamin A. Following termination of kinase reactions, proteins were resolved by SDS-PAGE. Signal from incorporation of ^32^P was detected by exposure to a phosphorscreen (top panel), and total protein was detected by Coomassie brilliant blue staining (bottom panel). The positions of radiolabeled GST-UL97 (GST97) and lamin A, and Coomassie stained lamin A are indicated. (The amounts of GST-UL97 were too small to see on the stained gel.) (B) UL97 autophosphorylation and labeling of lamin A were quantified following in *in vitro* kinase reactions in the presence of varying concentrations of maribavir (MBV). Signal detected from ^32^P incorporation for autophosphorylation of GST-UL97 and phosphorylation of His-tagged lamin A are plotted as a percent of the signal detected in the absence of drug. The results taken together show that UL97 phosphorylates lamin A in vitro.

We wished to determine whether UL97 can directly phosphorylate the largest of the A-type lamins, lamin A, in vitro. We expressed lamin A fused to a histidine tag at its N-terminus, and purified it from *E. coli* (Figure S2). When incubated with purified enzymatically active UL97 fusion protein (GST-UL97) and γ-[^32^P]-ATP, both GST-UL97 and lamin A became radiolabeled (Figure 1A). However, labeling of lamin A was not observed in the absence of added enzyme, and was almost completely abolished when an equivalent quantity of a catalytically deficient mutant form of GST-UL97 (K355Q) was used in place of wild-type GST-UL97 (Figure 1A). Moreover, treatment with maribavir, a specific inhibitor of UL97 activity, reduced phosphorylation of lamin A by GST-UL97 with a dose-dependence very similar to its inhibition of autophosphorylation of GST-UL97 (Figure 1B). Thus, UL97 phosphorylates lamin A *in vitro*.

**Figure 2:**
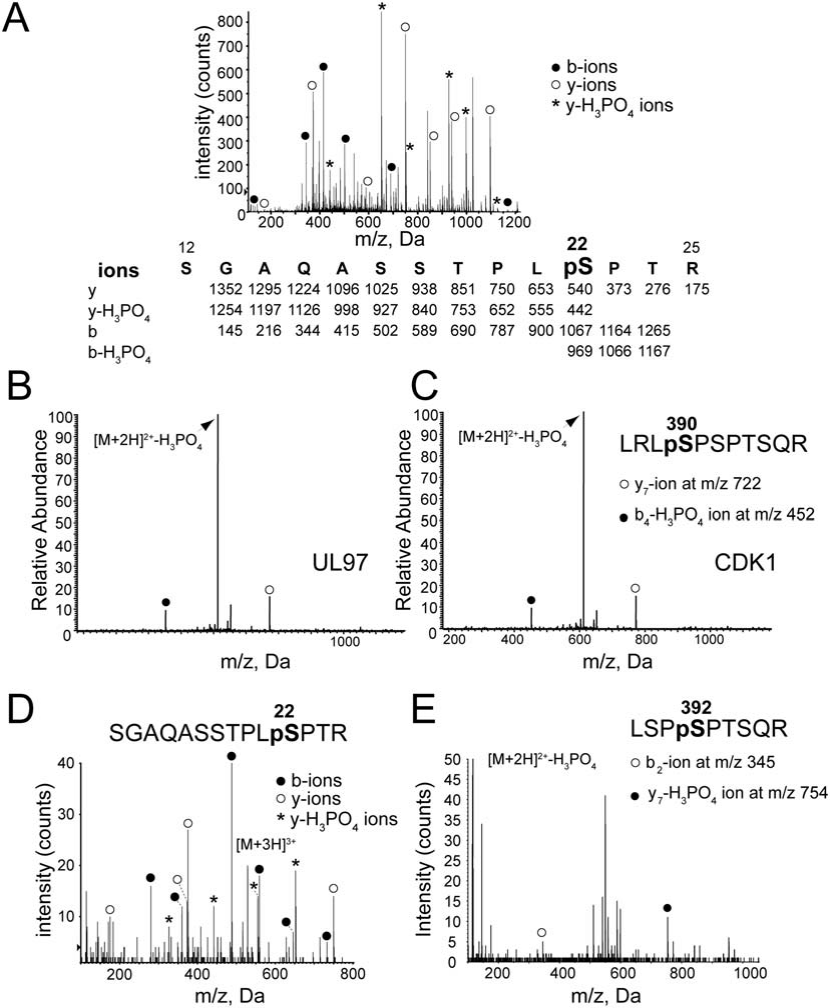
Phosphorylation sites detected on lamin A/C from HCMV-infected cells and from UL97 treated lamin A in vitro. (A) Mass spectrum from electrospray ionization (ESI)-MS-MS of SGAQASSTPLpSPTR tryptic peptide (amino acids 12–25 of native lamin A) after phosphorylation of His-lamin A in vitro with GST-UL97 indicating phosphorylation at Ser^22^. Diagnostic fragment ions used to verify detection of the sequence and the phosphorylation site are indicated in shaded circles and asterisks. A diagram of predicted fragment ions is shown below the spectrum. (B) Mass spectrum from ESI-MS-MS of LRLpSPSPTSQR tryptic peptide (amino acids 386–397 of native lamin A) after phosphorylation of His-lamin A in vitro with GST-UL97, indicating phosphorylation at Ser^390^. Diagnostic fragment ions are indicated as above. (C) Mass spectrum from ESI-MS-MS of LRLpSPSPTSQR tryptic peptide (amino acids 386–397 of native lamin A) after phosphorylation of His-lamin A in vitro with recombinant human CDK1/cyclin B complex, indicating phosphorylation at Ser^390^. Diagnostic fragment ions are indicated as above. (D) Representative mass spectrum from ESI-MS-MS of SGAQASSTPLpSPTR tryptic peptide (amino acids 12–25 of native lamin A) after immunoprecipitation of lamin A/C from HCMV-infected cells and iTRAQ labeling. Diagnostic fragment ions are presented as above. Note that the iTRAQ label alters the mass of the b-ions. The masses of the ions from this spectrum are presented in Figure S3. (E) Representative mass spectrum from ESI-MS-MS of LSPpSPTSQR tryptic peptide (amino acids 389–397 of native lamin A) after immunoprecipitation of lamin A/C from HCMV-infected cells. Diagnostic fragment ions are indicated as above. The results show that UL97, like CDK1 phosphorylates lamin A on Ser^22^ and Ser^390^ in vitro, but that phosphorylation occurs on Ser^392^ (and Se^r22^) in infected cells.

To map the sites on lamin A that are phosphorylated by UL97 *in vitro*, proteins from kinase reactions, such as those conducted in Figure 1A — except using unlabeled ATP — were digested with trypsin. Phosphopeptides were enriched, and the peptides analyzed by LC-MS/MS. Two major phosphorylated peptides on lamin A were reproducibly detected following phosphorylation by GST-UL97. One of these peptides (residues 12–25) was unambiguously phosphorylated on Ser^22^, a site whose phosphorylation by CDK1 is crucial for lamin disassembly [5]. A representative MS/MS spectrum showing Ser^22^ phosphorylation in tryptic peptides released from lamin A incubated with GST-UL97 is presented in Figure 2A. A second major peptide including Ser^390^ and Ser^392^ was clearly phosphorylated, but in the initial analysis there was ambiguity as to which of these serines was phosphorylated (see further analysis below). Three other phosphorylated peptides– one containing Ser^414^, one Ser^628^, and one Ser^652^ (Table 1) — were detected more than once, but less frequently than the two major peptides. No phosphopeptides were reproducibly detected in reactions using GST-UL97 K355Q or in reactions containing GST-UL97 and 1 µM maribavir (data not shown).

**Table 1:** A-type lamin phosphopeptides detected by mass spectrometry.

Position	Sequence	*in vitro* (UL97)	Infected cells
Ser22	SGAQASSTPLpSPTR	+	+
Ser390	LRLpSPSPTSQR	+	−
Ser392	LSPpSPTSQR	−	+
Ser403-Thr409	ApSpSHpSpSQpTQGGGSVTK^a^	−	+
Ser414	ASSHSSQTQGGGpSVTK	+	−
Ser423/Thr424	KLEpSpTESR^b^	−	+
Ser628	pSVGGSGGGSFGDNLVTR	+	−
Ser652	SYLLGNSpSPR	+	−

A-type lamin phosphopeptides detected after treatment with UL97 *in vitro* or detected in HCMV infected cells are indicated with a **+**. Those that were found in one setting, but not the other are indicated with a **−**.

a Singly phosphorylated peptides with phosphorylation at Ser403, Ser404, Ser406, Ser407, or Thr409 were each detected.

b Phosphorylation could not be confidently assigned between Ser423 or Thr424.

In order to definitively assign the site of *in vitro* phosphorylation by GST-UL97 on the second major peptide, we used an isotopically labeled synthetic peptide phosphorylated at Ser^392^ to identify fragment ions diagnostic for this phosphorylation site. As an additional control, we also treated lamin A with a commercial preparation of CDK1/cyclin B_1_ complex. For both GST-UL97 and CDK1/cyclin B_1_ treated samples of recombinant lamin A, no phosphopeptides matching the fragmentation pattern for phosphorylation at Ser^392^ were detected. Instead, a tryptic peptide (387–397) with phosphorylation at Ser^390^ was detected (Table 1, Figure 2B and 2C). Evidently, phosphorylation at Ser^390^ by UL97 or by CDK1 inhibited tryptic cleavage at Arg^388^. Possible reasons why we observed CDK1 phosphorylation on Ser^390^ rather than the more generally accepted site, Ser^392^ [7] include folding of the *E. coli* expressed lamin A altering the *in vitro* site specificity of CDK1. Reexamination of the original study [7] suggests that the data presented are consistent with a mixture of peptides; the peptide reported, which is phosphorylated at Ser^392^, and the longer peptide that we detected, which is phosphorylated at Ser^390^. Interestingly, CDK1 mediated phosphorylation of murine A-type lamins at Ser^390^ *in vitro* has been previously suggested [25]. Regardless, UL97 phosphorylates lamin A in vitro on two sites, Ser^22^ and Ser^390^, that are phosphorylated in vitro by CDK1 (Figure 2, data not shown, and [5],[6],[7]).

### UL97-dependent phosphorylation of lamin A/C at Ser^22^ during HCMV replication

To examine the role of UL97 in lamin A/C phosphorylation during HCMV infection, we infected cells with wild type (wt) HCMV strain AD169 at an MOI of 1, and at 72 hours post-infection (p.i.) prepared cell lysates from which we immunoprecipitated lamin A/C. We then subjected the lamin A/C immunoprecipitates to tryptic digestion and LC-MS/MS to examine the pattern of phosphorylation (Table 1). Ser^22^ phosphorylation was again detected (Figure 2D provides an example from an iTRAQ labeling experiment — see below). A phosphopeptide containing either Ser^390^ or Ser^392^ was also detected, but in this case, with the aid of the isotopically labeled synthetic peptide phosphorylated at Ser^392^, the site of phosphorylation was unambiguously identified as Ser^392^ (Figure 2E). The longer tryptic peptide containing Ser^390^ that was observed following in vitro phosphorylation was not detected in the lamin A/C immunoprecipitates. Four other phosphorylation sites were also detected (Ser^403^, Ser^406/407^, Ser^423^, Thr^424^), which are either identical or in close proximity (Ser^404^) to sites previously detected in studies of phosphorylated proteins from human cells not infected by HCMV [26],[27],[28],[29].

As mutational analysis implicates Ser^22^ and to a lesser extent Ser^392^ in lamin A/C disassembly [5], we employed an iTRAQ™-based quantitative MS approach [30] to evaluate whether levels of phosphorylation on these Ser residues were influenced by UL97 during HCMV replication. Cells were infected at an MOI of 1 with wt HCMV or a *UL97* deletion mutant, Δ97 [31] or a virus in which the deletion mutation was rescued [31] and lamin immunoprecipitates were prepared at 72 hours p.i. Following digestion with trypsin, peptides from each sample were labeled with a sample-specific iTRAQ reagent. Stable isotopes incorporated into these reagents permit pooling of samples after labeling and subsequent relative quantification of phosphopeptide abundances by LC-MS/MS. In two separate experiments, while levels of Ser^392^ phosphorylation did not vary between lamin A/C isolated from wt-infected vs. Δ97-infected cells, there were 2–3 fold higher levels of phosphorylation of Ser^22^ in lamin A/C from wt HCMV-infected cells compared to lamin A/C from Δ97-infected cells (Table 2). Furthermore, lamin A/C from cells infected with the virus in which the deletion was rescued also showed approximately 2-fold higher levels of Ser^22^ phosphorylation compared to that from cells infected with Δ97 (Table 2). Another pair of previously described mutant and rescuant viruses was also compared, one encoding a catalytically-deficient form of UL97 (AD K355Q), and the other a rescuant of AD K355Q, in which sequences encoding a wild-type UL97 were restored (AD Q355K) [22]. In this experiment, lamin A/C from cells infected with the rescuant (AD Q355K) had 4.1-fold higher levels of Ser^22^ phosphoryation than that from AD K355Q-infected cells (Table 2). From these results, we conclude that there is UL97-dependent phosphorylation of lamin A/C at Ser^22^ during HCMV infection.

**Table 2:** Comparison of lamin A/C phosphorylation at positions Ser22 and Ser392 during replication of wild-type and *UL97* mutant HCMVs.

Position	Phosphopeptide abundance			
	AD169rv/Δ97		FLAG97/Δ97	AD Q355K/AD K355Q
	Experiment 1	Experiment 2		
Ser22	2.3	3.0	1.8	4.1
Ser392	0.9	0.9	1.1	1.4

The ratios of abundances of the indicated phosphopeptides of lamin A/C from cells infected for 72 hours (MOI = 1) with the indicated pairs of viruses were determined using iTRAQ labeling and LC-MS/MS.

### Transient inhibition of UL97 decreases lamin A/C phosphorylation during viral replication

**Figure 3:**
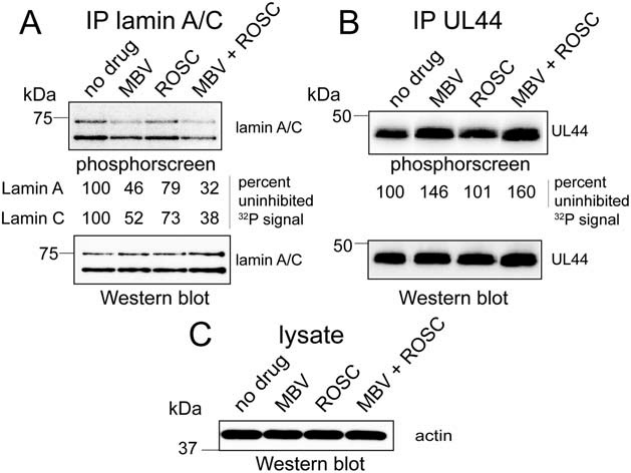
Orthophosphate labeling of lamin A/C during HCMV infection. HCMV-infected cells (MOI = 3, 69 hours p.i.) were pulse labeled for 2 hours with [^32^P]-orthophosphate in the presence or absence of maribavir or roscovotine or both and then lysed. (A) Lamin A/C immunoprecipitates were prepared, resolved by SDS-PAGE, and transferred to a PVDF membrane. The membrane was exposed to a phosphorscreen (top panel, phosphorscreen) to detect ^32^P signal, and then probed with anti-lamin A/C antibodies to detect total lamin A/C in the immunoprecipitates (bottom panel, western blot). The signal from the phosphorscreen was quantified and the values for ^32^P incorporation into lamin A and lamin C are shown between the upper (phosphorscreen) and lower (western blot) panels. (B) HCMV UL44 immunoprecipitates were prepared from lysates of the same radiolabeled infected cells, resolved by SDS–PAGE and transferred to a PVDF membrane. The membrane was exposed to a phosphorscreen (top panel, phosphorscreen) to detect ^32^P signal, and then probed with anti-UL44 antibodies antibodies to detect total UL44 in the immunoprecipitates (bottom panel, western blot). The signal from the phosphorscreen was quantified and the values for ^32^P incorporation into UL44 are shown between the upper (phosphorscreen) and lower (western blot) panels. (C) The lysates used for immunoprecipitation in A were resolved by SDS-PAGE and transferred to a PVDF membrane that was probed with anti-actin antibodies. Abbreviations: IP, immunoprecipitate; MBV, maribavir; ROSC, roscovitine. The results show that transient inhibition of UL97 with maribavir reduces phosphorylation of lamin A/C by about 50%.

As a second approach to investigating the dependence of lamin A/C phosphorylation on UL97, we performed radiolabeling experiments with wt HCMV infected cells (MOI = 3) in the presence or absence of 1 µM maribavir, which specifically inhibits UL97 [23],[32], or 15 µM roscovitine, which specifically inhibits CDKs including CDK1 [33],[34]. To minimize the effects of kinase inhibition on earlier stages of the HCMV replication cycle, infection was allowed to proceed for 69 hours p.i., at which time nuclear egress has commenced. The infected cells were then treated with the inhibitors or mock-treated with DMSO-containing vehicle for 1 hour, and then, in the presence or absence of the inhibitors, labeled with [^32^P]-orthophosphate for 2 hours. Following radiolabeling, cells were lysed. Probing western blots of the lysates with anti-actin antibodies showed that similar amounts of protein were present in each lysate (Figure 3C), Lamin A/C was immunoprecipitated from each lysate, the immunoprecipitates were resolved using SDS-PAGE, and transferred to a membrane. ^32^P-incorporation into lamin A/C in each sample was measured by exposing the membrane to a phosphorimager. Then, the membrane was probed with anti-lamin A/C antibodies to assess the amounts of lamin A/C in each immunoprecipitate. Similar amounts of lamins A and C were detected in each sample (Figure 3A, lower panel); however, there was somewhat more lamin A and C in the samples from cells treated with maribavir or maribavir plus roscovitine than in the mock-treated sample. Despite that, the incorporation of radioactive phosphate into lamin A and C was visibly less in the sample from infected cells treated with maribavir compared to mock-treated infected cells, (Figure 3A, upper panel). Phosphorimager quantification indicated that the reduction in labeling was approximately 2-fold. In contrast, the CDK inhibitor roscovitine had a less pronounced effect on lamin A/C phosphorylation, with radioactive phosphate incorporation reduced by 20–30% compared to the DMSO treated control (Figure 3A, upper panel). When both drugs were present, radioactive phosphate incorporation was reduced by 60–70% (Figure 3A, upper panel). (Again, there was more lamin A/C, as detected by immunoblotting, in the sample treated with both drugs than in the mock-treated control; thus, the actual effect of the drugs was likely even greater.)

As an additional control, in the same labeling experiment HCMV UL44 was immunoprecipitated, the immunoprecipitates were resolved by SDS-PAGE and transfered to a membrane, and both the amounts of UL44 and the incorporation of ^32^P into UL44 were measured on the same membrane. In this case, the overall incorporation of radioactive phosphate into HCMV UL44 was not reduced by treatment with maribavir, in fact, it was increased (Figure 3B, upper panel), while the amounts of UL44 were very similar in all the samples (Figure 3B, lower panel). (Further studies of UL44 phosphorylation have shown that UL97 phosphorylates only one or a few sites on UL44, while other kinases phosphorylate a number of other sites robustly (unpublished results).) Thus, transient treatment with maribavir exerted a greater inhibitory effect than transient treatment with a CDK inhibitor on lamin A/C phosphorylation, while not inhibiting the overall phosphorylation of a control protein, UL44.

### UL97 is required for alterations in the nuclear lamina during HCMV replication

**Figure 4:**
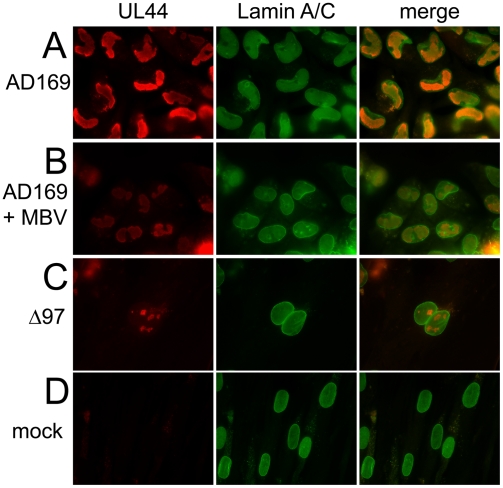
Immunofluorescence of lamin A/C in cells infected with HCMV. At 96 hours p.i. (MOI = 1), cells were fixed and double-stained with a mouse monoclonal antibody against UL44, a replication compartment marker (red, left panels) and a goat antibody against lamin A/C (green, center panels). Signals from both antibodies were merged (right panels). (A) Wild-type HCMV strain AD169 (AD169) infected cells show characteristic deformation of the nuclei. The nuclei of cells infected with AD169 in the presence of 5 µM maribavir (AD169+MBV) (B) or in cells infected with UL97 deletion mutant virus RCΔ97 (Δ97) (C) retain the oval shape characteristic of mock-infected cells (mock) (D).

Morphological deformations of cell nuclei are common in cells that are defective for lamin A/C [35]. Infection by HCMV has long been known to cause distortions in nuclear shape [36], and another study has reported that UL97 mutants do not induce such deformations [37]. Given our identification of lamin A/C as a substrate for UL97, we investigated whether these distortions were associated with alterations in the nuclear lamina. Therefore, we examined nuclear morphology during infection (MOI = 1) by staining the nuclear lamina with a polyclonal antibody against lamin A/C, while co-staining for sites of HCMV DNA replication (replication compartments) using a monoclonal antibody against the viral DNA polymerase subunit, UL44 (Figure 4). In immunofluorescence images of mock-infected cells (Figure 4D), the nuclei stained with the lamin antibody and were oval-shaped with no anti-UL44 staining. In wt HCMV-infected cells at 96 hours p.i. (Figure 4A), anti-lamin A/C staining revealed deformed nuclei, many of which exhibited a kidney shape. This shape was mirrored by those of the replication compartments stained with anti-UL44. Wt-infected cells treated with the UL97 inhibitor maribavir (Figure 4B) and cells infected with a *UL97* null mutant virus (RCΔ97) (Figure 4C) showed much less dramatic shape changes, and while nuclei were observed to be somewhat enlarged relative to mock-infected cells, they often retained an oval shape. In addition, the anti-lamin staining appeared more uniform around the rim of the nuclei in mutant-infected cells, maribavir-treated wt-infected cells, or mock-infected cells than in wt-infected cells (compare Figure 4A to 4B, 4C and 4D). These kinds of changes in shape and anti-lamin staining could be observed in wt HCMV-infected cells by 24 hours p.i., although less consistently (data not shown).

**Figure 5:**
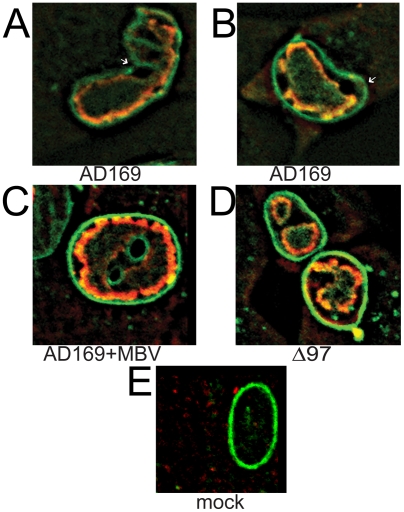
Confocal microscopy of UL97-dependent nuclear lamina alterations in HCMV-infected cells. At 96 hours p.i. (MOI = 1), cells were fixed and double stained for UL44 (red) and lamin A/C (green) and imaged by confocal microscopy. Discontinuities in lamin A/C staining were detected in wt AD169-infected cells (A) and (B) and are labeled with white arrows. Lamin A/C staining was more uniform and intense around the nuclear rim and gaps in staining were rarely observed in cells infected with AD169 in the presence of maribavir (MBV) (C), when a *UL97* deletion mutant virus was used (Δ97) (D), or in mock-infected cells (E).

**Figure 6:**
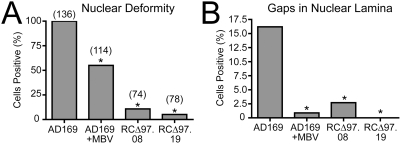
Quantification of UL97-dependent nuclear lamina perturbations during HCMV infection. (A) Occurrence of nuclear deformity in fibroblasts infected with HCMV strain AD169 (AD169), AD169 in the presence of maribavir (AD169+MBV), and independent isolates of UL97 deletion mutant RCΔ97 (RCΔ97.08) and (RCΔ97.19). Asterisk denotes Fisher's exact test *p*-value of <0.001 compared to AD169. Numbers above bars indicate numbers of cells scored. (B) Occurrence of gaps in the nuclear lamina in the same panel of cells scored above for nuclear deformity. Asterisk denotes Fisher exact test *p*-value of <0.001. These results show that UL97 is required for nuclear deformation and disruptions of lamina during HCMV infection.

We then used the same antibodies and confocal microscopy to investigate these staining patterns and their requirement for active UL97 in more detail. In wt HCMV-infected cells (MOI = 1; 96 hours p.i.), we observed thinning, invaginations (as recently reported [17]), and, in some cells, discrete gaps in anti-lamin staining (Figure 5A and 5B) compared to mock-infected cells (Figure 5E). In contrast, when wt HCMV infection was performed in the presence of maribavir or when *UL97* was ablated genetically (two independent isolates of a *UL97* deletion mutant), the anti-lamin staining of the nuclear rim resembled that of mock-infected cells with oval shaped nuclei and little or no thinning and few or no gaps (Figure 5C and 5D). To quantify the UL97-dependent changes in nuclear morphology, we surveyed approximately one hundred nuclei from each infection condition by confocal microscopy. These results indicated that nuclear deformities (Figure 6A) and gaps in the nuclear lamina large enough to be visible by light microscopy (Figure 6B) were each significantly more frequent under conditions where UL97 kinase activity was present (*p*<0.001). Thus, UL97 is required for nuclear deformation and disruptions of lamina during HCMV infection.

## Discussion

We found that UL97 can directly phosphorylate lamin A/C *in vitro* on sites phosphorylated *in vitro* by Cdk1, including Ser^22^. We also detected UL97-dependent phosphorylation of Ser^22^ on lamin A/C in HCMV-infected cells. Additionally, phosphorylation of lamin A/C was reduced by approximately two-fold when UL97 kinase activity was transiently inhibited in HCMV-infected cells. These findings taken together strongly suggest that lamin A/C is a bona fide, direct substrate of UL97 in infected cells.

Moreover, we found that UL97 is required in infected cells for discrete changes in the nuclear lamina, including gaps visible by confocal light microscopy. Given our results on lamin phosphorylation by UL97, we propose a model in which UL97 phosphorylates lamin A/C on Ser^22^, a site whose phosphorylation is known to mediate disassembly of nuclear lamina [5],[7]. These phosphorylation events contribute to localized disruptions in the nuclear lamina. These disruptions permit access of HCMV nucleocapsids to the inner nuclear membrane for primary envelopment and budding into the space between the inner and outer nuclear membranes, and thus nuclear egress. This model explains the requirement for UL97 for efficient nuclear egress [19].

We now discuss our results in terms of this model and previous results regarding lamin phosphorylation and nuclear egress.

### UL97 mimics CDK1 for direct phosphorylation of lamin A/C

UL97 directly phosphorylates *E. coli* expressed lamin A *in vitro* with Ser^22^ and Ser^390^ being the major sites phosphorylated. Neither of the major sites conforms to a preference of UL97 for basic residues in the P+5 position [32], although the sites do contain basic residues at P+6 and P+7. (A minor site (Ser^414^) phosphorylated by UL97 has an Arg at P+5.) More importantly, the major sites phosphorylated by UL97 were also phosphorylated in vitro by CDK1/cyclin B_1_. This result and previous results with Rb [22] indicate that UL97 can exhibit at least some preference for sites that CDKs prefer.

### UL97-dependent phosphorylation of lamin A/C at Ser^22^ in infected cells

We observed UL97-dependent phosphorylation in infected cells of a site that UL97 phosphorylates directly in vitro, and we found that transient inhibition of UL97 with maribavir could inhibit lamin A/C labeling by ∼50%. The simplest interpretation of our data is that UL97 directly phosphorylates lamin A/C in HCMV-infected cells. An alternative interpretation is that UL97 acts indirectly, for example, by increasing the activity and/or expression of host cell kinases that phosphorylate lamin A/C, in particular CDK1. However, if UL97 were acting indirectly to increase lamin A/C phosphorylation by increasing CDK activity or expression, then roscovitine should have been at least as effective at inhibiting lamin phosphorylation as maribavir. Our finding that maribavir was more effective than roscovitine is consistent with UL97 directly phosphorylating lamin A/C rather than increasing the activity or expression of a CDK. This interpretation is also consistent with a study that found that expression of a dominant negative CDK1 did not adversely affect replication of HCMV [38]; if CDK1 were required for disruption of nuclear lamina during nuclear egress, such expression might have been expected to decrease HCMV replication.

Although only one site phosphorylated by UL97 *in vitro* showed dependence on UL97 for its phosphorylation in virus infected cells, it is striking that this position — Ser^22^ — is the lamin A/C phosphoacceptor site of greatest established relevance to dissolution of lamina [5]. In particular, substitution of Ser^22^ with Ala results in a dominant negative mutant protein that inhibits lamina disassembly, while substitutions of Ser^392^ exert little effect on lamina disassembly by themselves [5]. Similarly, substitution of lamin B at Ser^16^, the position equivalent to Ser^22^ of lamin A/C, renders lamin polymers resistant to phosphorylation-mediated disassembly, while substitutions of serines equivalent to Ser^390^ and Ser^392^ have little or no effect [8]. Therefore, our results together with these previous studies strongly suggest that UL97 phosphorylation of lamin A/C in infected cells at Ser^22^ drives lamin A/C disassembly.

We also detected UL97-independent phophorylation of lamin A/C in our infected cell preparations. This could be due to the activities of other kinases in infected cells, due to phosphorylated lamin A/C that existed prior to infection, and/or due to phosphorylation occurring in uninfected cells in our samples.

### Relationship of UL97 effects on lamins to nuclear egress

In previous studies, it was reported that wt HCMV infection resulted in drastic reductions in the amounts of A-type lamins detected on western blots [20],[39], and almost complete loss of lamin staining as detected by immunofluorescence [20], but that neither was observed during infection by a UL97 mutant [20]. However, we observed copious expression of A-type lamins during wt HCMV infection, and, similar to the recent report of Camozzi et al. [17], we observed only discrete changes in lamin staining in immunofluorescence experiments. The failure of others to detect lamin A/C in infected cells may be due to using antibodies that fail to recognize hyperphosphorylated A-type lamins, as previously asserted [20], or some other form of lamin A/C. Additionally, it has been reported that expression of UL97 following transient transfection induces a redistribution of lamin A/C from the nuclear rim to granular intranuclear structures [20],[40]. Whether this was due to lamin A/C phosphorylation or due to toxicity (e.g. apoptosis) following UL97 overexpression was unclear. Regardless, we showed here that UL97 is required for disruptions in the nuclear lamina in HCMV-infected cells.

The simplest explanation for this requirement is that limited phosphorylation of lamins by UL97 causes local disassembly of the lamina. Although we detected gaps visible by confocal light microscopy in only a minority of cells, we emphasize that such gaps are much larger than an HCMV nucleocapsid. We think it is highly likely that nearly all infected cells contain smaller gaps in the lamina that could readily permit access of HCMV nucleocapsids to the inner nuclear membrane during nuclear egress. It may be advantageous for HCMV to induce only localized disruptions of the nuclear lamina. Lamins are important for cellular chromatin organization, DNA synthesis and transcription [3],[35], and thus may play important roles in herpesvirus infection in addition to their service as barriers for nuclear egress. Indeed, during HSV infection, lamin A/C is required for proper targeting of the viral genome and for reduction of heterochromatin formation on viral promoters at early times of infection [41]. Nevertheless, there is more extensive disruption of the nuclear lamina during HSV infection than what we observed during HCMV infection [42],[43].

The requirement for UL97 for the characteristic kidney-shape of nuclei in HCMV-infected cells is consistent with the results of Azzeh et al. [37]. The concave invaginations found in these nuclei are typically adjacent to juxtanuclear structures that appear to be cytoplasmic sites of virion assembly [44], and whose morphology depends on UL97 [37]. It is possible that the earliest UL97-mediated lamin disruptions (we detected gaps in some cells at 24 hours p.i. (data not shown) direct the passage of primary enveloped virions into a particular portion of the perinuclear cytoplasm, and this contributes to the organization of the juxtanuclear structure. Alternatively or additionally, UL97 may prevent aggregation of tegument proteins in the nucleus [45] or be more directly required for formation of the juxtanuclear structure [37]. As noted by Azzeh et al. [37], the juxtanuclear structure seems to impact the nucleus. Thus, it may exert the force that causes deformation of the lamin-depleted nuclear envelope.

A considerable body of evidence establishes that HCMV UL50 and UL53 and their homologs in other herpesviruses play important roles in nuclear egress (reviewed in [2]). It has been proposed that these proteins and their homologs alter the nuclear lamina by recruiting PKCs to phosphorylate lamins [15],[16],[17],[46],[47]. However, PKC phosphorylation of nuclear lamins normally occurs during interphase and is not sufficient to cause dissolution of lamina [6],[25]. Moreover, we did not observe phosphorylation of lamin A/C in HCMV-infected cells at sites known or likely to be phosphorylated by PKC, including Ser^5^, Thr^199^, Ser^395^, Thr^416^, Thr^480^, and Ser^652^ [7],[25]. It may be that UL50 and UL53 act by recruiting PKC to disassemble nuclear lamina, but that the relevant substrate of PKC is lamin B [48] or other nuclear envelope components such as emerin [49],[50]. Alternatively, the roles of UL50 and UL53 during nuclear egress may be independent of protein kinase recuitment. Recently, Camozzi et al. [17] reported that transient overexpression of HCMV UL50 and UL53 was sufficient to induce changes in the distribution of lamin A/C akin to what is observed during HCMV infection. Regardless, during HCMV infection, in the absence of UL97, nuclei remain oval and lamin staining remains intact despite the presence of UL50 and UL53. Thus, much as CDK1 is the crucial kinase for dissolution of lamina during mitosis, UL97, which mimics CDK1 for phosphorylation of lamin A/C, is the crucial kinase for nuclear egress.

### Why mimic CDKs?

Why has HCMV evolved to encode a kinase that mimics CDK1 for phosphorylation of lamin A/C? One possible explanation stems from HCMV arresting the cell cycle at the G1/S boundary [9],[10],[11]. CDK1 is most active in phosphorylating nuclear lamina during M-phase [6]. There are elevated levels of CDK1 and cyclin B_1_ in HCMV-infected cells [12],[13],[14], which may account for the phosphorylation of Ser^22^ and Ser^392^ on lamin A/C that we detected even in the absence of UL97. It is also possible that this phosphorylation contributes to disruption of the nuclear lamina. However, expression of a dominant negative CDK1 did not decrease HCMV replication [38], CDK1 and cyclin B do not appear to accumulate in the nuclei of infected cells to the extent seen in mitotic cells [14], and their elevated levels evidently are not sufficient to disrupt the nuclear lamina by themselves. Interestingly, CDK1 does appear to be required in HCMV-infected cells for the formation of so-called pseudomitoses, in which aberrant spindle poles and chromosome condensation occur [38], and CDKs that are sensitive to roscovitine are involved in several phases of HCMV replication [51].

UL97 mimicry of a CDK for phosphorylation of lamin A/C explains at least part of this viral enzyme's role in HCMV infection. It is notable that UL97 is also required for phosphorylation of a second CDK substrate, Rb, in infected cells [22],[52]. As yet, the importance of phosphorylation of Rb for HCMV replication has not been established. It will be interesting to elucidate further how HCMV uses its CDK-mimic to promote its propagation.

## Materials and Methods

### Reagents

All reagents were from Sigma unless otherwise specified.

### Cells and viruses

HFF cells, isolate Hs27, (American Type Culture Collection) were cultured in Dulbecco's modified Eagle's medium (DMEM) (VWR) supplemented with 10% fetal bovine serum (FBS) as described previously [31]. HCMV wt strain AD169 was obtained from the American Type Culture Collection. Two independent isolates of RCΔ97, RCΔ97.08 and RCΔ97.19, derived from AD169 and containing the *Escherichia coli lacZ* and *gpt* genes replacing most of UL97 [24], were generously provided by Mark Prichard (University of Alabama, Birmingham). In some experiments, AD169rv [53], a bacterial artificial chromosome (BAC) clone of HCMV strain AD169, as well as more recently constructed viruses derived from AD169rv, were used: Δ97, a BAC derived *UL97* deletion mutant, FLAG97 a rescuant of Δ97, AD K355Q, and AD Q355K [22],[31]. Viral stocks were propagated and titrated as previously described [31].

### Two-dimensional (2D) gel analysis of phosphoproteins

HFF cells were infected at a multiplicity of infection (MOI) of 1 PFU/cell (inoculum titers were confirmed by back titration) in complete medium (DMEM containing 5% FBS) for 2 h. Inocula were removed, and the cells were washed two times with complete medium. Complete medium, either with or without 1 µM maribavir, was then added and incubation was continued at 37°C. At 3 days post infection (p.i.), cells were rinsed twice with inorganic phosphate-free DMEM (Invitrogen) containing 1% FBS and were then incubated in 2 ml of that medium containing 1 mCi of [^32^P]-orthophosphate for 2 h. The medium was removed, and the cells were rinsed twice in ice-cold Tris-buffered saline (TBS: 20 mM Tris HCl pH 7.5, 150 mM NaCl) and then scraped into ice-cold TBS. 2D gel electrophoresis was performed by isoelectric focusing (IEF) using Immobiline Dry Strips with a pH gradient from 3 to 10 (GE Healthcare Inc.) in the first dimension and 4 to 20% (Invitrogen) SDS-PAGE in the second dimension as described [54]. Proteins were transferred for 30 minutes at 20 V to a BioTrace Polyvinylidene Fluoride (PVDF) membrane (Pall Corporation, Pensacola, Florida), such that approximately half of the protein was transferred and half remained in the gel. ^32^P signal from proteins transferred to PVDF was visualized by exposure to a phosphorscreen or Bio-Max Film (Kodak), and remaining protein in the gel was stained with Gel Code Blue colloidal coomassie blue staining reagent (Pierce) and was kept for subsequent MS analysis.

### Protein expression and purification

A pET19b vector (EMD Chemicals, Inc.) which was modified to incorporate a human rhinovirus 3C protease cleavage site following the pET19b polyhistidine tag, a generous gift from Tapan Biswas (Harvard Medical School), was used to express lamin A in *E. coli*. The lamin A sequence was amplified by PCR with an upstream primer (5′CCC*CATATG*ATGGAGACCCCGTCCCAG3′) containing a NdeI site, and a downstream primer (5′TTG*CTCGAG*TCATGATGCTGCAGTTCTG3′) containing a XhoI site (restriction sites are in italics), from plasmid pJB311 [55], kindly provided by Joel Baines (Cornell University). The resulting plasmid was used to transform *E. coli* BL21 (DE3) CodonPlus bacteria (Stratagene), and expression was induced at an optical density of 595 nm of 0.5, protein expression was induced with 0.5 mM isopropyl-β-D-thiogalactopyranoside (IPTG) for 20 h at 16°C. Inclusion bodies were isolated and dissolved in 8 M urea, 50 mM Tris-HCl pH 8.0, 0.5 M NaCl, 1 mM dithiothreitol (DTT), 20 mM imidazole and Complete™ protease inhibitor (Roche, 1 tablet/50 ml). The protein was then purified using a nickel column (Amersham Pharmacia) using the same urea buffer with a 20 to 1 M imidazole gradient. Proteins were concentrated and stored frozen and, when required were renatured by dialysis against 0.5 M NaCl, 50 mM Tris-HCl pH 8, 1 mM DTT and protease inhibitors.

GST-UL97 (WT) and GST-UL97 K355Q were expressed from baculovirus vectors as previously described [56], except that in some experiments, newly generated baculoviruses were used to express GST-UL97 and GST-UL97 K355Q fusion proteins. These baculoviruses were based on the Bac-to-Bac system (Invitrogen, Inc., Carlsbad, California) and derived from customized pFASTBAC transfer vectors incorporating an N-terminal glutathione S-transferase tag. Protein concentrations were determined by amino acid analysis at the Molecular Biology Core Facility, Dana-Farber Cancer Institute.

### Protein kinase assays

For radioactive *in vitro* kinase assays, 32 ng of GST-UL97 or GST-UL97 K355Q was used with 0.6 µg of His-lamin A per 20 µL reaction in 50 mM Tris (pH 8.5 at 37°C), 125 mM NaCl, 5% glycerol, 10 mM MgCl_2_, 2 mM DTT, 5 mM betaglycerophosphate, 100 µM unlabeled ATP and 0.5 µL of [γ-^32^P]-ATP (3,000 to 6,000 Ci/mmol) (Perkin Elmer Inc., Waltham, MA) and incubated at 37°C for 90 min. For non-radiactive kinase reactions submitted for MS analysis, reactions were scaled up to 120 µL and 2 µg of UL97 or 160 ng of CDK1/cyclin B complex (Cell Signaling Technology, Inc., Danvers, MA) was used, and the final ATP concentration was adjusted to 200 µM. For comparison of maribavir inhibition of UL97 autophosphorylation versus lamin A phosphorylation, the same reaction conditions were used except 50 ng GST-UL97, 0.5 µg of His-lamin A, 50 mM Tris (pH 9.0 at 25°C), 300 mM NaCl, 1 mM DTT, 10 mM MgCl_2_, 0.5 ml of [γ-^32^P]-ATP (3,000 to 6,000 Ci/mmol) (Dupont NEN) and specified concentrations of maribavir were combined in a final volume of 10 µl and incubated at 37°C for 2 h. Reactions were terminated by the addition of concentrated SDS-PAGE loading buffer. Samples were heated at 95°C for 5 min and proteins were resolved by SDS-PAGE. Gels were dried onto blotting paper under vacuum, and incorporated ^32^P was quantified with a phosphorimager (Molecular Imager FX System; Bio-Rad Laboratories Inc., Hercules, California).

### Immunoprecipitation and western blotting

For immunoprecipitations of lamin A/C for MS analysis, HFF were infected at an MOI of 1.0 (confirmed by back-titration). At 72 h post infection, cells were rinsed. In non-radioactive experiments, cells were rinsed twice in ice-cold Dubecco's phosphate buffered saline prior to lysis. For radioactive experiments, cells were rinsed three times in 0.5 mL of ice-cold Tris-buffered saline (25 mM Tris pH 8.0, 4 mM KCl, 137 mM NaCl) prior to lysis. Cells were lysed for 30 min at 4°C in 0.5 mL of ice-cold modified radio-immunoprecipitation assay (RIPA) buffer per well. The RIPA buffer used was similar to one previously described [57] except that 300 mM NaCl was used, Triton-X 100 (1%) was used in place of NP-40, and the following components were added: 10 mM EDTA, 10% glycerol, 10 mM betaglycerophosphate, 5 mM NaF, 10 µg/mL each of leupeptin and aprotinin, 1 µg/mL pepstatin A, 10 µM E-64, 2 mM imidazole, 1.2 mM sodium molybdate, 0.5 mM sodium orthovanadate, 4 mM sodium tartrate, 1 mM DTT, and Calbiochem Phosphatase Inhibitor Set I (EMD Chemicals Inc., Gibbstown, New Jersey) was added at 1∶100. Lysates were collected and clarified at 10,000 *g* at 4°C and supernatants were transferred to new tubes, flash frozen on liquid N_2_, and stored at −80°C until use.

Two hundred microliters of each thawed lysate was pre-cleared, with rotation, for 30 min at 4°C with 10 µg each of purified mouse IgG_1_ and IgG_2a_ (Bethyl Labs Inc., Montgomery, Texas) and 20 µL of settled Immunopure Protein A/G (Pierce) in a final volume of 0.5 mL (adjusted by adding RIPA buffer). Then, 220 µL of each sample was added to 15 µL of Protein A/G, which had been pre-incubated with 10 µL JOL2 and 5 µL JOL4 mouse anti-lamin A/C monoclonal antibodies (Millipore Inc., Billerica, Massachusetts). IP reactions were adjusted to a final volume of 0.5 mL with RIPA buffer and allowed to rotate for 1 h at 4°C. IP reactions were then subjected to four 5 min washes in 0.5 mL RIPA buffer and then incubated at 85°C for 5 min in 80 µL of 2× SDS-PAGE sample buffer [57] supplemented with 5% betamercaptoethanol. Immunoprecipitation of lamin A/C from non-radiolabeled cells was performed essentially as above except without pre-clearing.

For immunoprecipitation of HCMV UL44, each sample of cells was lysed in 1 ml EBC2 lysis buffer (50 mM Tris [pH 8.0], 30 mM NaCl, 0.5% NP-40, 2 mM EDTA and 2 mM DTT) supplemented with 10 µg/mL each of leupeptin and aprotinin, 1 µg/mL pepstatin A, 25 mM betaglycerophosphate, 0.5 mM sodium orthovanadate, and 1∶100 of Phosphatase Inhibitor Cocktail 1 and 2 (Sigma). Lysates were pre-cleared then reacted with anti-UL44 antibody 28-21 (kindly provided by William Britt, U. of Alabama) pre-conjugated to Protein A beads. The immunoprecipitations were washed four times in 1 mL cold EBC2 lysis buffer, and resuspended in 40 µL 2× SDS-PAGE sample buffer.

SDS-PAGE and western blotting procedures were carried out as previously described [58] using goat anti-lamin A/C polyclonal antibody N-18 (Santa Cruz Biotechnology), anti-CMV ICP36 (UL44) monoclonal antibody-ViruSelect (Virusys), or anti-beta-actin mouse monoclonal antibody (Abcam) to probe immunoblots.

### MS analysis

In early experiments, following SDS-PAGE, lamin A from in vitro phosphorylation reactions or lamin A/C from infected cells in gel slices was reduced and alkylated by DTT and iodoacetamide, respectively. The gel slices were then dehydrated in acetonitrile, and the protein digested with trypsin (500 ng/slice) in NH_4_HCO_3_ buffer (50 mM, 50 µL/slice) overnight at 37°C. Trypsin-digested peptide samples were then enriched for phophopeptides using a phophopeptide isolation kit (Pierce). The samples were mixed with binding buffer (10% acetic acid), followed by addition of sample mix (50 µL) to SwellGel Disc (Pierce) resin. The sample-resin mixture, whose pH was maintained below 3.5, was incubated for 8 minutes at room temperature with regular gentle swirling. Resin was washed twice in 50 µL of 0.1% acetic acid, and twice in 50 µL of 0.1% acetic acid, 10% acetonitrile. Phosphopeptides were eluted in elution buffer (50 µL, 100 mM ammonium bicarbonate, pH 9.0) after 5 minutes incubation. Eluted phophopeptides were analyzed by LC-MS/MS at the Taplin Biological Mass Spectrometry Facility at Harvard Medical School.

Subsequent experiments were performed using different protocols depending on whether phosphorylation was assessed following in vitro phosphorylation or following phosphorylation in infected cells. For analyses of in vitro phosphorylated samples, the kinase reactions were treated with 1 µg trypsin (Promega) in 100 mM ammonium bicarbonate pH 8.0 (overnight, 37°C), and lyophilized. Free peptide carboxylate groups were converted to their corresponding methyl esters and the derived peptides were subjected to phosphopeptide enrichment as described previously [59]. Captured phosphopeptides were eluted with 50 mM PO_4_ buffer pH 8.0 directly onto a 100 µm (i.d.)×8 cm fused silica capillary desalting precolumn (PC) packed with 10/25 irregular-shaped C18 beads. The PC was then connected to a 50 µm×8 cm (5 µm spherical C18 beads) analytical column. LC-MS/MS was performed on a QSTAR XL (MDS SCIEX). The HPLC solvent gradient was 0–7% B in 5 min, 7–63% B in 30 min; solvent A was 0.2 M acetic acid and solvent B was 70% acetonitrile/0.2 M acetic acid.

In later experiments aimed at comparing the phosphorylation sites of UL97 and CDK1 on lamin A/C, in vitro phosphorylated lamin A/C was digested as described above and each digest was analyzed separately on an LTQ-Orbitrap mass spectrometer (ThemoFinnigan) using a method consisting of two data-dependent MS/MS scans, followed by targeted MS/MS scans on precursors corresponding to lamin A/C peptides containing Ser^22^, Ser^392^, their phosphorylated counterparts, and a synthetic pS392 peptide (LSPpSPTSQR) containing 6^13^C and 4 ^15^N atoms. This synthetic peptide was loaded onto the PC as an internal control.

For analysis of lamin A/C phosphorylated in cells, lamin A/C immunoprecipitates was processed for subsequent iTRAQ (Applied Biosystems) isotope labeling according to the manufacturer's protocol. Briefly, each sample was separately reduced using 5 mM Tris-(2-carboxyethyl)phosphine (TCEP) (1 hr, 60°C), alkylated using 10 mM methyl methane-thiosulfonate (MMTS) (10 min, room temperature) and digested on the beads with trypsin (Promega) in 0.5 M triethylammonium bicarbonate pH 8.5 (overnight, 37°C). The resulting peptides were then labeled with iTRAQ-114 (mock), iTRAQ-115 (AD K355Q), iTRAQ-116 (Δ97) and iTRAQ-117 (AD169rv) reagents in 70% ethanol, respectively, for 1 hr at room temperature. Following iTRAQ labeling, all four samples were combined and dried by centrifugal concentration (Thermo Savant, Holbrook, New York). To enrich for phosphorylated lamin A/C peptides, 100 µl of 100 mg/ml MassPREP Enhancer in 80% acetonitrile/0.1% trifluoroacetic acid (TFA) were added. After sonication (20 min), the mixed sample was loaded on a well of a TiO_2_ 96-well plate (Waters, Milford, Massachusetts). Peptides were eluted with 100 µl of 300 mM ammonium hydroxide. Following elution, 3 µl of TFA were added to the sample to bring the pH to 2.0 and the sample volume was reduced to ∼20 µl by centrifugal concentration. LC-MS/MS was performed on a QSTAR XL using a Top 3 method. A similar protocol was followed for analysis of AD169rv- (iTRAQ 114), Δ97-(iTRAQ 115), FLAG97 (WT rescue of Δ97)- (iTRAQ 116), mock-(iTRAQ 117), AD K355Q-(iTRAQ 114) and AD Q355K-(iTRAQ 115) HCMV-infected cells, except that a targeted MS/MS method (on phosphorylated Ser^22^ and Ser^392^) was used for the phosphopeptides and a Top 6 was used for the TiO_2_ loading flowthrough. Non-phosphorylated lamin peptides detected in the loading flowthrough were used to normalize abundances of phosphorylated peptides. As described above, an isotopically labeled synthetic phosphopeptide containing a phosphate on Ser^392^ was loaded onto the PC as an internal standard.

MS/MS spectra were searched against an in-house lamin A/C database and the human RefSeq database using the Mascot algorithm. Putative lamin A/C phosphopeptide sequences were manually confirmed.

### Transient inhibition of protein kinase activities in cells

2×10^5^ HFF per well of a 24 well cluster plate were infected at a MOI of 3 (confirmed by back titration) and then incubated at 37°C in DMEM supplemented with 5% FBS. Sixty-nine hours post infection, medium was removed and cells were washed with phosphate-free DMEM (Invitrogen Inc.) and incubated for 1 hour in 0.5 mL phosphate free medium containing 2% FBS, supplemented with 15 µM roscovitine or 1 µM maribavir, both drugs, or no drug. The concentration of DMSO was adjusted to a final concentration of 0.2% (vol/vol) to control for any effects of the carrier. Five hundred microcuries of ^32^P-labeled orthophosphoric acid (8500–9120 Ci/mmol) was then added in 0.5 mL of phosphate free medium containing 2% FBS and the same drug condition used during the pre-incubation step and left on the cells for 2 h. Lamin A/C and HCMV UL44 were immunoprecipitated as described above and analyzed by SDS-PAGE and autoradiography and western blotting as described above.

### Immunofluorescence

HFF were seeded for at 1×10^5^ cells/well on glass coverslips in 24-well dishes and allowed to attach overnight prior to infection at an MOI of 1. At 96 h post infection, cells were fixed in 4% formaldehyde in PBS for 20 min (unless otherwise indicated, all steps were performed at room temperature). Cells were permeabilized with acetone at −20°C for 2 min. Following several washes in PBS, cells were blocked overnight in IF buffer (PBS containing 4% FBS [Sigma]). Primary antibodies were diluted in IF buffer and incubated with fixed cells for 1 h. Lamin A/C goat polyclonal antibody N-18 (Santa Cruz Biotechnology, Inc.) was used at 1∶10 dilution and UL44 mouse monoclonal antibody 10-C50 (Fitzgerald Industries International Inc.) was used at 1∶100. Secondary antibodies (Alexa Fluor 568-rabbit anti-mouse IgG and Alexa 488-chicken anti-goat IgG, Invitrogen) were applied at 1∶1,000. Cells were then washed in IF buffer three times for 5 min per wash, and mounted on slides in Prolong Antifade reagent (Molecular Probes, Inc.). Fluorescence microscopy was performed in the Nikon Imaging Center at Harvard Medical School, using a Nikon TE2000U inverted microscope in conjunction with a PerkinElmer Ultraview spinning disk confocal system equipped with a Hamamatsu Orca ER cooled-CCD camera. Images were acquired using a 60× differential interference contrast oil immersion objective lens and analyzed using Metamorph software from Universal Imaging, Inc. (Downingtown, Pennsylvania). Fisher's exact test was performed using Prism 4 (GraphPad Software, Inc.) for Macintosh.

## Supporting Information

Table S1 Candidate UL97 substrates. Protein description, database hit, and peptides detected for those polypeptides for which at least two distinct peptides were detected in one or more experiments are shown.
(0.05 MB DOC)

Figure S1 2D electrophoresis. Samples from HFF cells infected with HCMV wt strain AD169 in the absence of drug (left-most panels), UL97 null mutant RCΔ97 in the absence of drug (middle panels), and AD169 in the presence of 1 µM maribavir (MBV; rightmost panels) that had been radiolabeled with 32P orthophosphate were resolved by isoelectric focusing in one dimension (arrows above each panel point to lower pIs) and by SDS-PAGE in the second dimension (arrows to the left of the panels point to lower molecular weight (Mr). Top panels (A) show autoradiograms of the 2D gels; the red rectangles indicate the areas from which spots from wt-infected cells differed from those in the other two samples were taken. Bottom panels (B) show an expanded region of the gels in the top panels. Spots numbered 1–6 from wt-infected cells were excised from the gels and submitted for MS analysis. Lamin A/C peptides were found in spot 6.
(8.95 MB TIF)

Figure S2 Purification of recombinant lamin A. His-lamin A was expressed in *E. coli*, the bacterial cells were lysed and His-lamin A/C was purified as a fusion protein using nickel affinity chromatography as described in Materials and Methods. Aliquots of the initial crude protein extract derived from inclusion bodies (crude), the flow through, the first wash (wash 1), the second wash (wash 2), and the eluate (lanes indicated above the image of the gel) were resolved by SDS-PAGE alongside molecular weight markers (M) and detected by Silver Blue staining. The position of His-lamin A is indicated to the right of the gel. By this procedure, sufficient amounts of reasonably pure lamin A were obtained.
(0.03 MB PDF)

Figure S3 Diagram of predicted fragment ions from spectrum in Fig. 2D. The sequence of the peptide is shown on the top line, with the position of the iTRAQ label indicated. Below are the masses of the various series of ions.
(2.11 MB TIF)
