# Supplementary material for: Viral Mimicry of Cdc2/Cyclin-Dependent Kinase 1 Mediates Disruption of Nuclear Lamina during Human Cytomegalovirus Nuclear Egress
Source: PLoS Pathog. 2009 Jan 23;5(1):e1000275. doi: 10.1371/journal.ppat.1000275 (PMC2625439; doi:10.1371/journal.ppat.1000275)
Supplement: Table S1 — Candidate UL97 substrates. Protein description, database hit, and peptides detected for those polypeptides for which at least two distinct peptides were detected in one or more experiments are shown. (0.05 MB DOC) [file ppat.1000275.s001.doc]

**Supplementary Table S1. Candidate UL97 Substrates**

| Protein Description | Database Hit | No. of Peptides Detected |
| --- | --- | --- |
| Lamin A/C | LAMA_HUMAN | 16 |
| α-Enolase | ENOA_HUMAN | 15 |
| Cytokeratin 1 | K2C1_HUMAN | 10 |
| Tropomyosin 3 | TPM3_HUMAN | 9 |
| HCMV UL112/113 | EP84_HCMVA | 8 |
| HCMV UL83 (pp65) | PP65_HCMVA | 7 |
| Annexin I | ANX1_HUMAN | 7 |
| Aldehyde Reductase | ALDX_HUMAN | 7 |
| HCMV UL80/UL80.5 | VP40_HCMVA | 6 |
| Isovaleryl-CoA Dehydrogenase | IVD_HUMAN | 6 |
| Collagen-Binding Protein 2 | CBP2_HUMAN | 6 |
| Fructose-Bisphosphate Aldolase | ALFC_HUMAN | 5 |
| Elongation Factor 1-γ | EF1G_HUMAN | 5 |
| HCMV UL44 | VPAP_HCMVA | 4 |
| Acyl-CoA Thioester Hydrolase | CTE2_HUMAN | 4 |
| Elongation Factor 1-α-1 | EF11_HUMAN | 4 |
| Glyceraldehyde 3-Phosphate Dehydrogenase | G3P2_HUMAN | 4 |
| Tropomyosin 1 | TPM1_HUMAN | 4 |
| Tropomyosin 4 | TPM4_HUMAN | 4 |
| Annexin V | ANX5_HUMAN | 3 |
| Dead Box Protein 48 | DDX48_HUMAN | 3 |
| PCNA | PCNA_HUMAN | 2 |
| Tropomyosin 2 | TPM2-HUMAN | 2 |
| Galectin 1 | LEG1_HUMAN | 2 |
| Profilin 1 | PRO1_HUMAN | 2 |
| Rab GD1 β | GD1B_HUMAN | 2 |

Table Legend S1.

Protein description, database hit, and peptides detected for those polypeptides for which at least two distinct peptides were detected in one or more experiments are shown.
