# Supplementary figures and images for: Viral Mimicry of Cdc2/Cyclin-Dependent Kinase 1 Mediates Disruption of Nuclear Lamina during Human Cytomegalovirus Nuclear Egress
Source: PLoS Pathog. 2009 Jan 23;5(1):e1000275. doi: 10.1371/journal.ppat.1000275 (PMC2625439; doi:10.1371/journal.ppat.1000275)

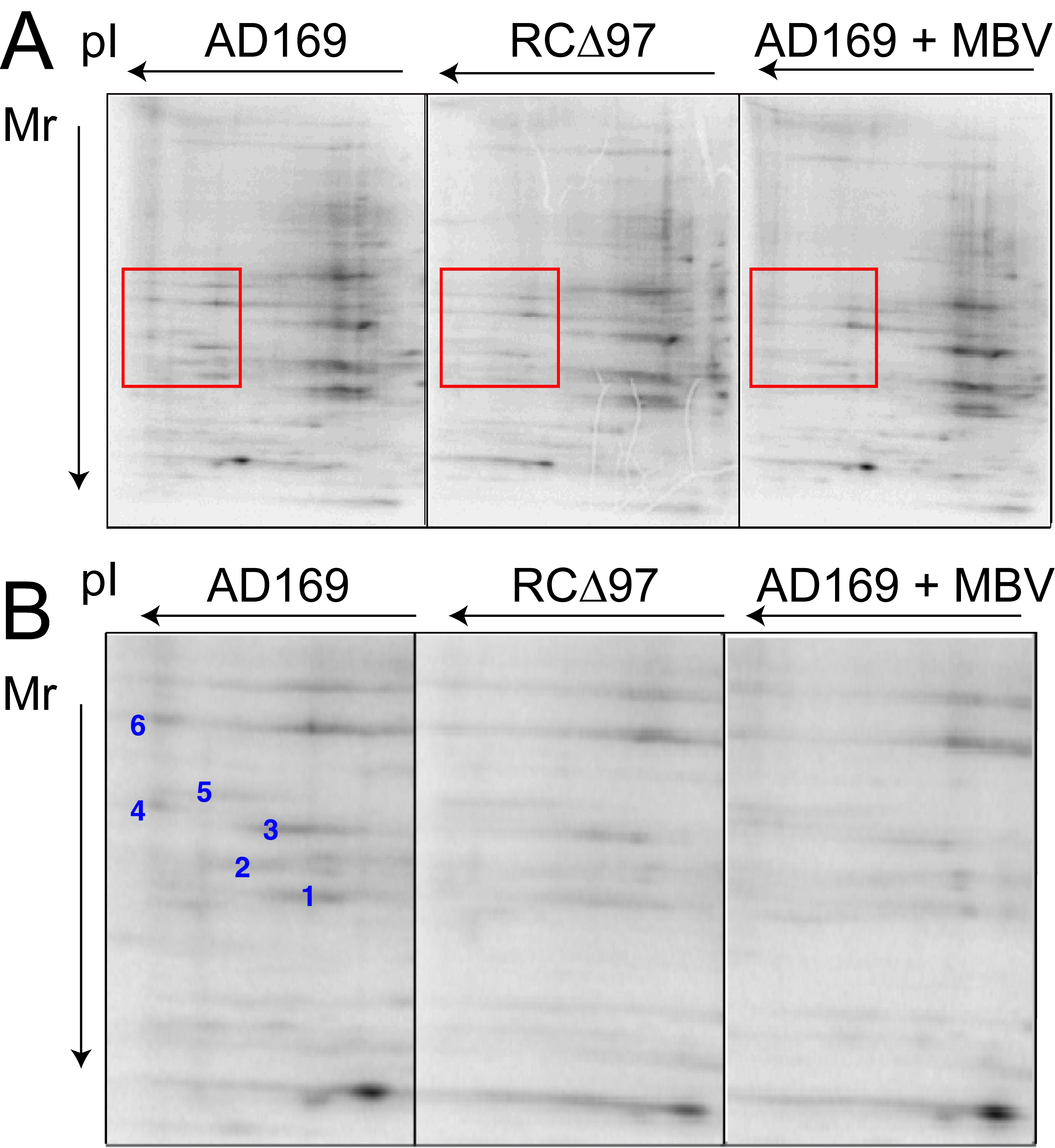

Supplement: Figure S1 — 2D electrophoresis. Samples from HFF cells infected with HCMV wt strain AD169 in the absence of drug (left-most panels), UL97 null mutant RCΔ97 in the absence of drug (middle panels), and AD169 in the presence of 1 µM maribavir (MBV; rightmost panels) that had been radiolabeled with 32P orthophosphate were resolved by isoelectric focusing in one dimension (arrows above each panel point to lower pIs) and by SDS-PAGE in the second dimension (arrows to the left of the panels point to lower molecular weight (Mr). Top panels (A) show autoradiograms of the 2D gels; the red rectangles indicate the areas from which spots from wt-infected cells differed from those in the other two samples were taken. Bottom panels (B) show an expanded region of the gels in the top panels. Spots numbered 1–6 from wt-infected cells were excised from the gels and submitted for MS analysis. Lamin A/C peptides were found in spot 6. (8.95 MB TIF) [file ppat.1000275.s002.tif]

M      crude      flow through      wash 1      wash 2      eluate

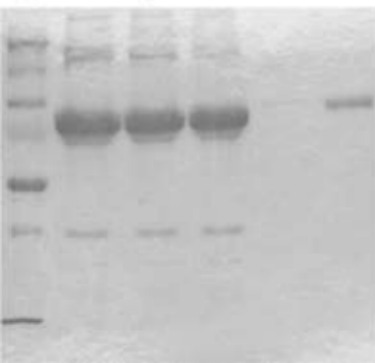

← lamin A

Supplement: Figure S2 — Purification of recombinant lamin A. His-lamin A was expressed in E. coli, the bacterial cells were lysed and His-lamin A/C was purified as a fusion protein using nickel affinity chromatography as described in Materials and Methods. Aliquots of the initial crude protein extract derived from inclusion bodies (crude), the flow through, the first wash (wash 1), the second wash (wash 2), and the eluate (lanes indicated above the image of the gel) were resolved by SDS-PAGE alongside molecular weight markers (M) and detected by Silver Blue staining. The position of His-lamin A is indicated to the right of the gel. By this procedure, sufficient amounts of reasonably pure lamin A were obtained. (0.03 MB PDF) [file ppat.1000275.s003.pdf]

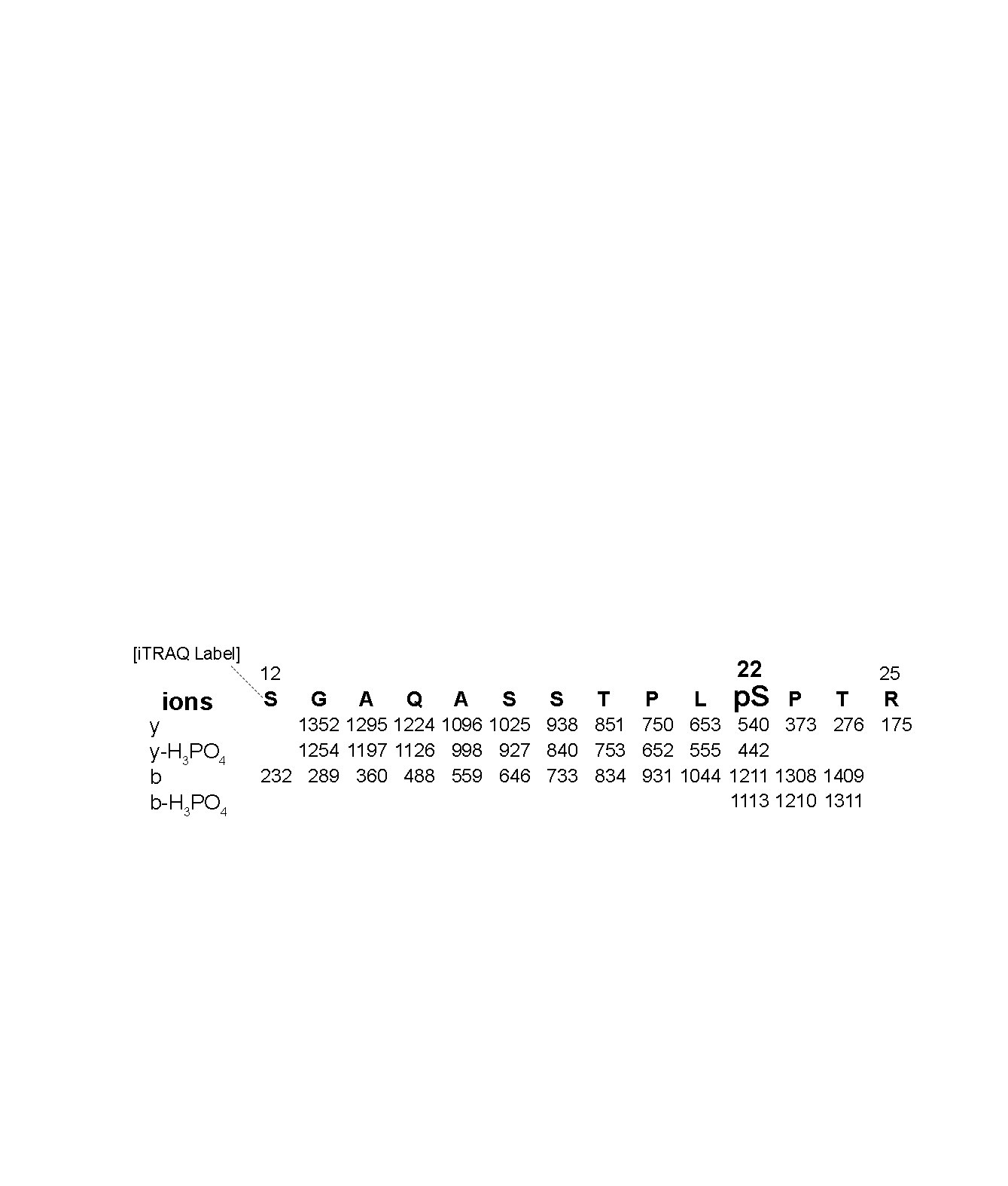

Supplement: Figure S3 — Diagram of predicted fragment ions from spectrum in Fig. 2D. The sequence of the peptide is shown on the top line, with the position of the iTRAQ label indicated. Below are the masses of the various series of ions. (2.11 MB TIF) [file ppat.1000275.s004.tif]
